# Hematopoietic stem cell transplantation: an Italian monocentric experience on the health assessment and eligibility of adult-related donors

**DOI:** 10.3389/fonc.2024.1389068

**Published:** 2024-05-30

**Authors:** Caterina Giovanna Valentini, Sara Ceglie, Federica Fatone, Elisabetta Metafuni, Claudio Pellegrino, Patrizia Chiusolo, Simona Sica, Luciana Teofili

**Affiliations:** ^1^Dipartimento di Scienze di Laboratorio ed Ematologiche, Fondazione Policlinico Universitario “A. Gemelli” IRCCS, Rome, Italy; ^2^Sezione di Ematologia, Dipartimento di Scienze Radiologiche ed Ematologiche, Università Cattolica del Sacro Cuore, Rome, Italy

**Keywords:** related donor, donor assessment, eligibility criteria, hematopoietic stem cell transplantation, engraftment

## Abstract

**Introduction:**

Indications for HSCT are increasing worldwide, paralleled by a growing demand for donors of therapeutic cells.

**Methods:**

Herein, we report our real-world experience of adult HPC donor assessment during a 5-year study period (2018–2023): we have retrospectively revised data of 455 potential related stem cell donors, consecutively evaluated at our center. Donor medical history was assessed by a questionnaire and an interview with a trained physician experienced in donation procedures to evaluate donor fitness and medical history. Pre-existing health disorders were fully investigated. Behavioral risk factors for communicable infectious diseases were also routinely explored.

**Results and discussion:**

Overall, 351 donors were finally assessed as eligible for HPC donation, and 233 underwent stem cell collection, 158 through apheresis from mobilized peripheral blood, and 75 through bone marrow harvest. Among them, 27 donors were selected despite the presence of pre-existing health conditions, which would be potential exclusion criteria for unrelated donors: 16 suffered from well-controlled cardiovascular diseases (CVD) and 11 from allergic diathesis. Most of the selected donors with pre-existing disorders were candidates for apheresis HPC collection (21, 77.8%), while only six (22.2%) underwent BM harvest. We then analyzed the data relative to the corresponding 233 allogeneic HSCT to explore if the presence of pre-existing diseases in the donors could show any association with transplant characteristics. Transplants from CVD and allergy donors showed no significant disparities in comparison with those from healthy donors. A significant difference emerged regarding the disease severity, with a higher proportion of patients with high/very high disease risk index (DRI) among those receiving grafts from CVD donors (68.7% in transplants from CVD donors versus 36.0% in transplants from healthy donors, p=0.005). Multivariate analysis confirmed that high/very high DRI patients had an increased probability of receiving donations from CVD donors (OR, 4.89; 95%CI, 1.15–20.86; p=0.031). Among donors with well-controlled pre-existing conditions, no adverse events were recorded during stem cell collection or at follow-up. Our results suggest that in patients at high risk for relapse requiring a prompt allogeneic transplant, a familiar donor might be accepted for HPC apheresis donation on less strict criteria than unrelated donors, without risk for both donor and patient.

## Introduction

Allogeneic hematopoietic stem cell transplantation (allo-HSCT) is an established treatment for a wide range of acquired or congenital disorders (1–4). At present, available stem cell sources include bone marrow (BM), mobilized peripheral blood (PB), and cord blood. Indications for HSCT are increasing worldwide, paralleled by a growing demand for donors of therapeutic cells. The practice of haploidentical HSCT, together with the advances in transplant technique, supportive care, and conditioning regimens, allows the treatment of older patients with elderly familial donors or donors with comorbidities, imposing different challenges to hematologists (5, 6). Pretransplant hemopoietic progenitor cells (HPC) donor assessment and testing are critical processes that affect the quality and safety of donation. Many issues on donor safety have been addressed in recent years, but most data are collected from unrelated donors, while consistent information from related donors is sparse (7). For unrelated donors, detailed recommendations for the health assessment have been published (8, 9), allowing HPC donations only if they are in good health, without any medical conditions. The donors must have a performance status that permits safe apheretic HPC collection or be able to tolerate anesthesia during BM harvest, with adequate cardiac, pulmonary, hepatic, and renal function. Eligibility criteria for related donors are less rigorous and may vary between centers. In 2015, the Worldwide Network for Blood and Marrow Transplantation (WBMT) Standing Committee developed a consensus document with recommendations for donor workup and final clearance of family donors who were not eligible as unrelated donors because of their age or pre-existing diseases (10). The document has been recently updated (11). Despite that specific diagnosis and/or disease severity directly imply the donor exclusion, the presence of other well-controlled conditions may be overcome and does not prevent per se the donation.

Herein, we report our real-world experience of HPC donor assessment during a 5-year study period (January 2018–October 2023); we have retrospectively revised anamnestic data of potential related stem cell donors, consecutively evaluated at our center. The study aims to explore if the presence of one or more pre-existing health disorders in donors deemed eligible according to the recent WBMT recommendations (11) may have an impact on transplant outcomes.

## Methods

### Study population

We conducted a retrospective observational study, including potential related donors consecutively evaluated at the Transfusion Medicine Department of Fondazione Policlinico A. Gemelli IRCCS of Rome (Italy) and allo-HSCT consecutively performed from selected related donors at the Transplant Unit of the same hospital between 1 January 2018 and 31 October 2023. Transplants with BM grafts (HPC Marrow, HPC-M) and PB apheresis collections (HPC Apheresis, HPC-A) were included, while transplants with cord blood units were excluded from the study.

The study followed the tenets of the Declaration of Helsinki and received approval from the Ethics Committee of Fondazione Policlinico Universitario A. Gemelli IRCCS (Prot. 0030921/20).

### Donor assessment

All donors were qualified according to JACIE standards (12) and Italian National regulation for transfusion activities (13, 14).

Donor medical history was assessed by a questionnaire and an interview with a trained physician experienced in donation procedures, to evaluate donor fitness, medical history, and willingness to donate before HLA-typing. Pre-existing health disorders were explored to exclude inherited or genetic coexisting diseases and behavioral risk factors for communicable infectious diseases. Donors characteristics that resulted in deferral from HPC donation were grouped into 11 categories according to WBMT standards (11): 1) infectious disease, 2) autoimmune disorders (AID), 3) low weight (defined as a weight lower than 50 kg), 4) abuse of alcohol or drugs, 5) CVD), 6) promiscuous sexual activity or cohabitation with hepatitis carriers, 7) allergic diathesis, including drug allergies, 8) lack of proper venous access, 9) history of cancer, 10) recent history of major surgery, and 11) other conditions. CVD specifically comprise arterial hypertension, hypotension, coronary heart disease, disturbance of heart rate and rhythm, congestive heart failure, cardiomyopathy, valvular heart disease, pericarditis, myocarditis, atherosclerotic peripheral vascular disease, aortic aneurism, and cardiac surgery for congenital heart disease. According to the updated WBMT standards (11), donors affected by CVD are eligible for HPC apheresis collection if the American Society of Anesthesiology Physical Status (ASA-PS) classification (15) are ≤2, according to the European Society of Anaesthesiology (ESA) (16) and American College of Cardiology/American Heart Association guidelines (17). Among the autoimmune disorders, diseases with systemic multiorgan involvement are investigated such as ankylosing spondylitis, polymyositis, arteritis, dermatomyositis, polymyalgia rheumatic, arteritis, antiphospholipid antibody syndrome, multiple sclerosis/optic neuritis, systemic lupus erythematosus, scleroderma, Sjögren’s syndrome, vasculitis syndromes, and Behçet’s (11). Among single-organ autoimmune diseases, Hashimoto thyroiditis, Graves’ disease, pernicious anemia, psoriasis, alopecia areata, and vitiligo are included (11).

Laboratory tests were performed on HPC donors deemed potentially eligible according to their medical history and after collecting a medical interview. These tests included complete peripheral blood count, serum creatinine, electrolyte and liver function studies, coagulation test, thrombophilia screening, microbiological screening for communicable infections (serologic studies for cytomegalovirus, herpes viruses, syphilis, anti-HIV antibodies HIV RNA, hepatitis B and C viruses, including nucleic acid amplification testing) blood typing and red cell antibody screening, human leukocyte antigen (HLA) typing, chest X-ray, electrocardiography, and abdominal ultrasound.

All donors eligible for BM harvest underwent a preventive anesthesiological assessment, while for HPC-A donors, an evaluation of peripheral vascular access was requested.

### Mobilization protocol and apheresis procedures

Allogeneic peripheral blood stem cell (PBSC) donation was performed with subcutaneous (sc) administration of granulocyte colony-stimulating factor (G-CSF for days +1 to +4), followed by leukapheresis on day +5. G-CSF was administered at a standard dose of G-CSF 12 μg/kg sc daily. Prophylaxis with paracetamol was administered to prevent potential side effects of G-CSF. All collection procedures were performed at the Apheresis Unit using the COBE Spectra or Spectra Optia continuous flow cell separators (Terumo BCT, Shinagawa, Tokyo) with the mononuclear cell collection program, and a ratio anticoagulant: blood of 1:12. Anticoagulant always consisted of sodium citrate solution (Fresenius Kabi, Bad Homburg, Germany). In all patients, 2.5–3 total blood volumes (TBV, defined as the processing blood volume divided by the patient’s blood volume) were processed. The apheresis procedures aimed to achieve a CD34+ cell dose of 4.0x10^6^ per kg of the recipient’s body weight. If the required number of CD34+ cells/kg was not accomplished, G-CSF administration was continued, and a second collection procedure was performed on day +6. According to institutional procedures, in donors with circulating CD34+ cells <20/µL on day +5 or estimated collection harvest <1×10^6^/kg of the recipient’s body weight, sc plerixafor at the dose of 240 μg/kg/day was planned in the consecutive night, from 4 to 6 h before leukapheresis. Red blood cell depletion in major and bidirectional AB0 mismatched transplants, and plasma removal in minor AB0 mismatched transplants, or transplants with transfused donors or female donors with previous pregnancies, were performed as previously reported (18). From March 2020 onward, all HPC products were cryopreserved. This procedure was introduced due to the COVID-19 pandemic and is still in place (19).

### Bone marrow collection

BM harvest and processing were managed as previously reported (18, 20–22). The perioperative autologous donation practice was discontinued in April 2020, replaced by iron (ferric carboxymaltose 500 mg intravenous), and B12 vitamin supplementation (1 mg sc) 1 week before BM collection (22). BM was harvested from posterior iliac crests in the operating room under general anesthesia, with the goal to collect 20–22 ml/kg of the donor weight. After harvesting, donors were admitted to the Hematology Department and observed for the following 24–48 h or more, as necessary.

### Donor follow-up

All donors were followed for 1 year after the donation. Hematological and biochemical tests were routinely performed at 1 week, 6 months, and 12 months after donation.

### Donor, patient, and graft data

Donor variables included gender, age, weight, HLA match (HLA-identical, haploidentical, and 7/8 mismatch), and ABO match. Laboratory data included WBC and, for apheresis donors, CD34+ cell count in peripheral blood on the day of collection. Apheresis data included blood volume processed, content of total nucleated cells (TNC), and content of CD34+ cells in the apheresis product. Patient variables included basic demographics; diagnosis; date of transplant; disease status (complete remission or not), disease risk index (DRI) (23); hematopoietic cell transplantation comorbidity index (HCT-CI) (24); dates of neutrophil, platelet, and erythrocyte engraftment; the incidence of acute and chronic graft versus host disease (aGVHD and cGVHD, respectively) (25–27); relapse; and status at the last follow-up. Graft variables included source (BM or PBSC), TNC content, CD34+ cell content, and CD3+ cell content. Cell contents were expressed as cell dose (i.e., the number of cells per kilogram of the recipient’s body weight) and were obtained as previously reported (28).

### Study outcomes and definitions

We investigated the association of pre-donation health disorders with recipient features and transplant characteristics and outcomes, including patient’s age, HCT-CI, and DRI; conditioning regimens; neutrophil, platelet, and erythrocyte engraftment; all grade aGVHD and cGVHD; relapse; and recipient status at the last follow-up (alive or death). Neutrophil and platelet engraftments were defined as the achievement of an absolute neutrophil count (ANC) ≥ 0.5 × 10^9^/L and a PLT count ≥ 20 × 10^9^/L unsupported by transfusion, respectively; erythrocyte engraftment was defined as a reticulocyte count ≥ 2%. HCT-CI was defined according to Sorror et al. (24) DRI was defined according to Armand et al. (23) Diagnosis and grading of aGVHD and cGVHD were made according to standard criteria (25–27). Regimens were classified as myeloablative conditioning (MAC) or non-myeloablative (NMA) conditioning, including reduced-intensity conditioning (RIC) (18). In particular, MAC regimens consisted of fludarabine and total body irradiation (FLU-TBI; fludarabine 120 mg/m^2^, followed by 9–12 Gy TBI) or thiotepa, busulfan, fludarabine (TBF; thiotepa 5 mg/kg on day −6 and −5 total; intravenous busulfan 3.2 mg/kg on day −4, −3, and −2; fludarabine 50 mg/m^2^ on day −4, −3, and −2). Reduced and NMA regimens consisted of busulfan or fludarabine/TBI 2 Gy-based regimens.

### Statistical analysis

Continuous variables were expressed as median with relative interquartile range (IQR) and categorical variables as n (%). Univariate analysis of continuous variables was performed by the Mann–Whitney U test or the Wilcoxon rank test, as appropriate. For categorical variables, Fisher’s exact test or the χ^2^ test was used, as appropriate. The association between donor status (fully eligible or eligible despite pre-existing diseases) and patients or transplant characteristics was assessed by multivariate logistic regression analysis incorporating the donor status as the dependent variable and recipients and transplant variables as covariates. The results were expressed as odds ratio (OR) with the relative 95% CI. All tests were two-sided, and a p-value <0.05 was considered statistically significant. Missing data were always <5% and were not considered. Analyses were performed using the IBM SPSS Statistics 25.0 and NCSS 10 v 10.0.19.

## Results

### Donor eligibility evaluation

Overall, during the study period, we examined 455 potential HSC donors (ratio M/F 240/215), accounting for a median number of 1 donor for the patient (range, 1–7). The median age at first donation screening was 39 years (IQR, 30–51). **Table 1** summarizes the main characteristics of all evaluated donors.

**Table 1:** Characteristics of 455 potential HPC-related donors.

	N (%)
**Median screened donors/patient**	1 (1–2)
**Age, years**	39 (30–51)
**Males/Females**	240 (52.7)/215 (47.3)
**Weight, kg**	73 (62–84)
**Previous pregnancy/abortion**	121 (56.3)*
**ABO/Rh typing** ***O pos/neg*** ***A pos/neg*** ***B pos/neg*** ***AB pos/neg*** ***Not performed***	129 (28.4)/19 (4.2) 121 (26.6)/16 (3.5) 44 (9.7)/6 (1.3) 11 (2.4)/3 (0.6) 106 (23.3)
**Medical history**	
**Cardiovascular diseases** ***hypertension*** ***arrythmia*** ***congenital atrial septal defect*** ***pericarditis/myocarditis/endocarditis*** ***aortic aneurysm/ectasia*** ***hypotension*** ***carotid stenosis*** ***previous myocardial infarction***	44 (9.7) 24 (54.5) 9 (20.5) 3 (6.8) 3 (6.8) 2 (4.5) 1 (2.3) 1 (2.3) 1 (2.3)
**Autoimmune disorders** ***thyroiditis*** ***nonspecific antibodies positivity*** ***psoriasis*** ***coeliac disease*** ***rheumatoid arthritis***	31 (7.0) 22 (71.0) 3 (9.5) 2 (6.5) 2 (6.5) 2 (6.5)
**Allergic diathesis** ***drugs*** ***seasonal allergy*** ***perennial allergy*** ***nickel***	24 (5.3) 10 (41.7) 8 (33.3) 4 (16.7) 2 (8.3)
**Infectious diseases** ***HBV/HCV*** ***HPV*** ***EBV*** ***viral pericarditis/myocarditis*** ***tubercolosis*** ***syphilis*** ***malaria*** ***prion disease risk***	16 (3.5) 4 (25.0) 3 (18.8) 3 (18.8) 2 (12.5) 1 (6.3) 1 (6.3) 1 (6.3) 1 (6.3)
**Oncological diseases** ***thyroid*** ***skin*** ***colon*** ***breast*** ***lung*** ***uterus***	8 (1.8) 2 (25) 2 (25) 1 (12.5) 1 (12.5) 1 (12.5) 1 (12.5)
**Low weight**	5 (1.1)
**Abuse of alcohol or drugs**	6 (1.3)
**Promiscuous sexual activity or cohabitation with hepatitis carriers**	23 (5.1)
**Lack of proper venous access**	14 (3.1)
Recent history of major surgery	6 (1.3)
Others *medical investigations ongoing* *not allergic asthma* *diabetes mellitus II* *benign prostatic hypertrophy* *drugs* *recent pregnancy* *recent minor surgery/tattoo* *microcytic anemia* *underage*	25 (5.5) 5 (20.0) 2 (8.0) 1 (4.0) 1 (4.0) 6 (24.0) 2 (8.0) 6 (24.0) 1 (4.0) 1 (4.0)
Eligible donors	351 (77.1)

*Percentage calculated on female donors.

Continuous variables are given as median (interquartile range). Categorical variables are given as a number (%).

At donor assessment, one or more pre-existing health disorders were identified (**Table 1**). Among 455 donors, 16 (3.6%) were excluded for previous hepatitis B and C infection, 15 (4.7%) for promiscuous sexual activities or cohabitation with hepatitis carriers, eight (1.8%) for oncological diseases, six (1.3%) for a recent history of major surgery, and six (1.3%) for a previous history of alcohol or drug abuse. In addition, 14 donors (3.1%) were deferred from donation because of lack of peripheral venous access and five (1.1%) for a low body weight (< 50 kg). Overall, 31 donors (7% of the total), mainly female (26 F/5 M, 84% vs. 16%), were affected by one or more AID. Finally, 25 potential donors (5.5%) had several miscellaneous conditions that precluded HPC donation (**Table 1**).

Following our policy, we allowed HPC donation in donors with CVD if classified as ASA <3, in diabetes mellitus type 2 with no organ damage, or with allergic diathesis including drug allergies to a known pharmacological agent and nickel allergy. Accordingly, among 44 identified donors (9.7%) with CVD, 28 (63.6%) were definitively deferred from HPC donations, while 16 (36.3%) who were in well-controlled clinical conditions were deemed eligible for donation. Moreover, among 24 donors (5.3%) with allergic diathesis, 11 with allergies due to known pharmacological agents (9) or nickel allergy (2) were deemed eligible for donating.

Finally, after medical evaluation, 351 out of 455 donors were finally considered eligible for HPC donation and 233 underwent stem cell collection: 158 underwent apheresis and 75 bone marrow harvest. In total, among 233 selected donors, 27 had pre-existing well-controlled diseases, which would have prevented donation in the setting of unrelated HPC donors. **Table 2** details the main features of this group of related donors. Pre-existing CVDs were equally distributed in both female and male donors, while allergic diathesis was more frequent in women. CVD donors were more frequently aged >60 years, and they more frequently were HLA-identical siblings. Conversely, drug allergies were mostly reported in the haploidentical setting. Most part of selected donors with pre-existing disorders were candidates to apheresis HPC collection (21, 77.8%), while only six (22.2%) underwent BM harvest, and they were considered suitable to be exposed to general anesthesia (**Table 2**).

**Table 2:** Characteristics of 27 related donors with pre-existing well-controlled clinical conditions and 206 healthy ones.

	Healthy donors N=206 (%)	CVD donors N=16* (%)		Allergy donors N=11 (%)	
Age, years	56 (45–63)	56 (46.75–58.75)		37 (32–47)	
> 60 years	11 (5.4)	3 (18.7)		1 (9)	
Male/female	130/76 (63.1/36.9)	9/7 (56.5/43.8)		4/7 (36.4/63.6)	
Weight, kg	72 (62–83.3)	82.5 (68.5–94)		62 (55–91)	
Type of disorder	–	Hypertension	10 (62.5)	Drug allergy	9 (81.8)
	–	Hypotension	1 (6.3)	Nickel allergy	2 (18.2)
	–	Arythmia	1 (6.3)		
	–	Carotid stenosis	1 (6.3)		
	–	Atrial septal defect	1 (6.3)	–	–
	–	Aortic aneurysm	2 (12.3)	–	–
Previous pregnancy/abortion	40 (52.6)**	5 (71.4)**		6 (85.7)**	
ABO major/bidirectional	51 (24.8)	3 (18.8)		0	
HLA match					
*HLA-identical sibling*	75 (36.4)	10 (62.5)		4 (36.4)	
*Haploidentical sibling*	130 (63.1)	6 (37.5)		7 (63.6)	
*7/8*	1 (0.5)	0		0	
Stem cell source *PBSC* *BM*	137 (66.5) 69 (33.5)	14 (87.7) 2 (12.3)		7 (63.6) 4 (36.4)	

*Including one donor with diabetes mellitus type II.

**Percentage calculated on female donors.

CVD, cardiovascular diseases; HLA, human leukocyte antigen; PBSC, peripheral blood stem cell; BM, bone marrow. Continuous variables are given as median (interquartile range). Categorical variables are given as number (%).

### Graft collection

No adverse events were observed during mobilization or collection. Among 158 HPC-A donors, 141 (89.2%) underwent one single collection procedure, while in 17 cases (10.8%), a second collection procedure was performed to achieve the required CD34+ cell dose (**Table 3**). Moreover, two HPC-A donors received plerixafor the night between day+5 and day +6 after starting of G-CSF. Five donors (3.2%) received a low prophylactic dose of enoxaparin the evening before stem cell apheresis because of hereditary thrombophilia without a personal medical history of venous thromboembolism (two heterozygous factor V Leiden, two protein S deficiency, and one antithrombin III deficiency). Neither thrombotic nor hemorrhagic complications were subsequently observed. Regarding donors with pre-existing well-controlled medical conditions, only one apheresis donor with CVD experienced a second-day collection, while none received plerixafor or anticoagulant prophylaxis (**Table 3**). Similarly, no adverse events were recorded among BM donors.

**Table 3:** Characteristics of stem cell collections grouped according to the medical history of HPC-related donors.

	Healthy donors N=206 (%)			CVD donors N=16 (%)			Allergy donors N=11 (%)		
	BM 69 (33.5)	PBSC 137 (66.5)		BM 2 (12.3)	PBSC 14 (87.7)		BM 4 (36.4)	PBSC 7 (63.6)	
Total blood volume harvested (mL)	1,629.0 (13,821.5–1,749)	–		1,556.0 (1,516–1,597)	–		1,430.0 (1,207–1,639)	–	
Volume (mL/kg)*	20.5 (17.5–21.9)	–		21.8 (20.8–22.8)	–		21.3 (18.3–24.3)	–	
Two-day collection	–	16		–	1		–	0	
Inherited thrombophilia**	–	5		–	0		–	0	
Apheresis procedure	–	1st day collection	2nd day collection	–	1st day collection	2nd day collection	–	1st day collection	2nd day collection
TBV (L)	–	13.0 (11.0–14.0)	12.0 (10.3–13.0)	–	13.7 (11.7–14.3)	14.0	–	13.0 (11.0–14.0)	–
TBV (mL/kg)*	–	169.7 (143.3–200.0)	177.0 (15.2–216.7)	–	158.1 (141.1–200.0)	218.1	–	169.7 (143.3–200.0)	–
WBC (×10^9^/L) pre apheresis	–	46.90 (40.01–55.15)	50.62 (42.67–55.43)	–	48.99 (36.15–58.06)	54.24	–	46.88 (40.01–55.15)	–
CD34+ cells/µL pre apheresis	–	78.0 (54.2–103.0)	39.5 (31.0–59.8)	–	69.0 (54.5–94)	20.0	–	78.0 (54.2–103.0)	–

*Donor’s body weight.

**Heterozygous factor V Leiden (2), protein S deficiency (2), and antithrombin III deficiency (1).

CVD, cardiovascular diseases; PBSC, peripheral blood stem cell; BM, bone marrow; TBV, total blood volume processed; WBC, white blood count. Continuous variables are given as median (interquartile range). Categorical variables are given as a number (%).

### Donor follow-up

At follow-up, one healthy donor reported a hospital admission for cholecystectomy due to gallstones; the surgery occurred 3 months after the stem cell collection. No further adverse events were recorded among healthy donors or donors with pre-existing diseases.

### Recipients and transplants

Overall, 233 allo-HSCT were performed in 227 patients (male/female, 140/87). Six patients received a second HSCT due to graft failure in four cases and relapse in additional two cases. Diagnoses were acute myeloid leukemia and myelodysplastic syndromes (118 transplants), acute lymphoid leukemia (29 transplants), primary or post-myeloproliferative neoplasm myelofibrosis (45 transplants), Hodgkin’s and non-Hodgkin’s lymphomas, chronic lymphocytic leukemia, and multiple myeloma (34 transplants), and severe aplastic anemia (seven transplants).

**Table 4** illustrates the characteristics of recipients and transplants grouped according to the donor status (healthy donors, CVD donors, and allergy donors).

**Table 4:** Characteristics of 233 allogeneic stem cell transplantations, and results of univariate analysis comparing transplants from healthy donors with those from HPC-related donors with pre-existing medical conditions.

	Transplants from healthy donors N=206	Transplants from donors with CVD N=16	p-value*	Transplants from donors with allergy N=11	p-value**
Age, years	56 (45–63)	56 (46.7–58.7)	0.459	52 (40–62)	0.830
> 60 years	73 (35.4)	4 (25.0)	0.586	5 (45.4)	0.529
Male/female	130/76 (63.1/36.9)	8 (50.0)/8 (50.0)	0.600	7 (63.6)/4 (36.4)	0.109
Weight, kg	75 (65–85)	76 (61–85)	0.717	65 (60–75)	0.166
Diagnosis					
*Acute myeloid leukemia/MDS*	104 (50.5)	6 (37.5)	0.300	8 (72.7)	0.321
*Myeloproliferative neoplasms++*	39 (18.9)	6 (37.5)		0 (0.0)	
*Lympoproliferative disorder+*	32 (15.5)	1 (6.3)		1 (9.1)	
*Acute lymphoid leukemia*	25 (12.1)	3 (18.7)		1 (9.1)	
*Severe aplastic anemia*	6 (2.9)	0 (0.0)		1 (9.1)	
HCT-CI score > 2	116 (56.9)	9 (56.3)	0.794	8 (72.7)	0.362
DRI high/very high	71 (36.0)	11 (68.7)	0.005	5 (45.5)	0.503
Complete remission	98 (47.6)	8 (50.0)	1.000	5 (45.5)	1.000
ABO mismatch major/bidirectional	51 (24.7)	3 (18.7)	0.766	0 (0.0)	0.071
MA conditioning regimen *NMA conditioning regimen*	46 (22.3) 160 (77.7)	6 (37.5) 10 (62.5)	0.216	3 (27.3) 8 (72.7)	0.714
Female donor to male	42 (20.4)	2 (12.5)	0.744	3 (27.3)	0.701
PBSC graft BM graft	137 (66.5) 69 (33.5)	14 (87.5) 2 (12.5)	0.099	7 (63.6) 4 (36.4)	1.000
HLA match *7/8* *HLA-identical sibling* *Haploidentical sibling*	1 (0.5) 75 (36.4) 130 (63.1)	0 10 (62.5) 6 (37.5)	0.116	0 4 (36.4) 7 (63.6)	0.973
TNC × 10^8^/kg§	6.4 (4.5–8.6)	8 (6.0–9.4)	0.069	6.8 (4.8–7.7)	0.964
CD34+ cells × 10^6^/kg§	5.7 (3.8–7.5)	6.4 (2.8–7.8)	0.841	6.8 (3.9–8.3)	0.539
CD3+ cells × 10^6^/kg§	180.7 (43.6–266.1)	160.2 (80.0–252.5)	0.798	202.5 (39.3–247.6)	0.981
Neutrophil engraftment (days)	21 (17–25)	17 (17–22)	0.327	17 (16.75–21.75)	0.154
Platelet engraftment (days)	21 (15–29)	20 (16–33)	0.897	19 (14–24)	0.238
Erythrocyte engraftment (days)	30 (22–36)	23 (18–33.5)	0.265	25.5 (18.75–29.25)	0.197
Day +30 neutrophil engraftment	172 (83.9)	10 (62.5)	0.042	10 (90.9)	1.000
Day +30 platelet engraftment	138 (67.3)	8 (50.0)	0.176	9 (81.8)	0.508
Day +30 erythrocyte engraftment	90 (43.9)	6 (37.5)	0.794	7 (73.6)	0.227
aGVHD	42 (20.6)	2 (12.5)	0.744	1 (9.1)	0.697
cGVHD	37 (18.4)	1 (6.3)	0.694	1 (9.1)	0.693
Relapse	40 (19.4)	6 (37.5)	0.107	2 (18.2)	1.000
Death	69 (33.5)	9 (56.3)	0.099	3 (27.3)	1.000

MDS, myelodysplastic syndromes; + lymphoprolipherative disorders include Hodgkin lymphoma, non-Hodgkin lymphoma, chronic lymphocytic leukemia, and plasmacellular discrasias; ++ myeloproliferative neoplasms include idiopathic or post-myeloproliferative neoplasm myelofibrosis and myeloid chronic leukemia.

p* and p** indicate difference in comparison with other donations; § recipient’s body weight. Continuous variables are given as median (interquartile range). Categorical variables are given as number (%). Significant p-values are highlighted in bold.

CVD, cardiovascular diseases; HLA, human leukocyte antigen; PBSC, peripheral blood stem cell transplant; BM, bone marrow; HCT-CI, Hematopoietic Cell Transplantation-Comorbidity Index; DRI, Disease Risk Index; MA, myeloablative regimen; NMA, non-myeloablative regimen; TNC, total nucleated cells; MNC, mononucleated cells; aGVHD, acute graft versus host disease; cGVHD, chronic graft versus host disease. Continuous variables are given as median (interquartile range). Categorical variables are given as a number (%). Signiﬁcant values are in bold type.

Regarding transplants from CVD donors, no significant disparities were observed for baseline characteristics (patients’ age, diagnosis, conditioning type, donor type, graft type, and cell content). A significant difference emerged regarding the disease severity, with a higher proportion of patients with DRI > 2 among those receiving grafts from CVD donors (68.7% in transplants from CVD donors in comparison with 36.0% in transplants from healthy donors, p=0.005). There was no difference in the transplant cell doses, including TNC, CD34+ cells, and CD3+ cells (**Table 4**). Regarding transplants from allergy donors, no differences were found in comparison with transplants from healthy donors.

### Outcomes

Regarding engraftment, the median time to obtain neutrophils, platelet, and erythrocyte engraftment was similar in all patients, independently from the donor type (**Table 4**). However, a trend for a lower proportion of recipients with day-30 neutrophil engraftment was observed among transplants from CVD donors (62.5% in transplants from CVD donors in comparison with 83.9% in transplants from healthy donors, p=0.042) (**Table 4**). Moreover, similar proportions of patients in the three transplant groups developed aGVHD, cGVHD, or relapsed or died (**Table 4**).

Considering that the significant findings emerged only relative to transplants from CVD donors, we further evaluated in a multivariate regression logistic model the association between the CVD donor status and several recipient or transplant variables. We considered the CVD donor status as the dependent variable and included among covariates stem cell source, the CD34+ cell, and TNC graft content; HCT-CI>2; high/very high DRI; day-30 neutrophil; platelet and erythrocyte engraftment; and incidence of acute and chronic GVHD. We found that high/very high DRI patients had an increased probability of receiving donations from CVD donors (OR, 4.89; 95% CI, 1.15–20.86; p=0.031; **Table 5**).

**Table 5:** Multivariate analysis of the association of pre-existing well-controlled cardiovascular disorders in HPC donors’ medical history on patient conditions, graft cellular doses, and transplant outcomes.

	OR	95% CI	p-value
HCT-CI >2	0.57	0.14–2.38	0.444
DRI high/very high	4.89	1.15–20.86	0.031
Bone marrow source	0.49	0.04–6.09	0.581
CD34+ cells × 10^6^/kg§	1.18	0.87–1.59	0.284
TNC × 10^8^/kg§	1.05	0.83–1.32	0.704
Day-30 neutrophil engraftment	1.53	0.13–17.89	0.736
Day-30 platelet engraftment	0.85	0.16–4.63	0.850
Day-30 erythrocyte engraftment	1.03	0.24–4.43	0.964
aGVHD	1.13	0.20–6.48	0.888
cGVHD	0.33	0.03–3.29	0.347

§ Recipient’s body weight.

HCT-CI, Hematopoietic Cell Transplantation-Comorbidity Index; DRI, Disease Risk Index; TNC, total nucleated cells; aGVHD, acute graft versus host disease; cGVHD, chronic graft versus host disease. Significant p-values are highlighted in bold.

## Discussion

This study reported our monocentric experience in the health assessment of HPC-related donors and investigated for the first time the impact of pre-existing medical conditions in donors considered eligible on transplant outcomes. Our data showed that HPC collection can be safely performed in related donors suffering from some pre-existing diseases such as well-controlled cardiovascular disorders. In our setting, this situation occurred most for the transplant of patients at the highest risk for relapse, in whom allo-HSCT could not be postponed. Moreover, the presence of medical conditions that would have deferred donation in the unrelated setting, did not affect engraftment or was associated with an increased rate of acute or chronic GVHD.

The safety and welfare of the donor are recognized as major concerns for the transplantation community. Historically, HLA-matched sibling donors, available for 30% of patients, have been considered the best choice for both practical and biological reasons (29–31). Transplantation techniques have evolved over the past two decades. The practice of haploidentical HSCT, together with the advances in transplant technique, supportive care, and reduced-intensity conditioning regimens, allows the treatment of older patients with elderly familial donors or donors with comorbidities, imposing different challenges to hematologists. As a result of such substantial changes in the donation process, the best accessible graft for many patients may be from an older donor such as HLA identical-sibling or haploidentical relative, highlighting the importance of understanding if any characteristic of donors may negatively influence recipient outcomes. There are an increasing number of studies that have evaluated the risk associated with HPC collection in related donors (32–34), but no report has been published evaluating the impact of benign medical conditions of donors on transplant outcome.

Donor assessment and the final decision on donor clearance are under the responsibility of the collection center’s physicians. The evaluation of related and unrelated donors followed the same procedure based on the currently valid quality standards and recommendations [WMDA (35, 36), FACT-JACIE (37)]. Compared with the strict recommendations on the suitability of unrelated donors, criteria for related donors allow for more discretion. In 2015, the donor outcome committee of WBMT proposed consensus recommendations of suitability criteria for pediatric and adult-related donors, which have been recently updated (10, 11). The WBMT standards allow related donors with pre-existing health conditions that would have been deferred as unrelated donors might still undergo donation if these medical conditions are not expected to lead to a significant reduction in donor safety.

We excluded all potential donors with medical conditions or lifestyles that would have represented a serious risk for both the donor and the recipient. In our experience, 23% of related HPC donors would not fit the eligibility criteria of an unrelated donor registry because of pre-existing conditions, and medical history of autoimmune diseases, primarily thyroid affections, represented the main reason for non-eligibility. CVDs were the second cause of medical contraindications to HPC donation, mainly severe acute heart issues, uncontrolled hypertension, and tachyarrhythmias. Donors with ongoing malignancies or a history of a malignant condition other than minor skin cancers such as basal cell carcinomas were excluded from further consideration. As for eligible HPC donors, 12% would have been deferred according to WBMT standards (11), mainly because of hypertension. Of note, four of them were over 60 years of age. Similar results are reported from a Dutch study evaluating short- and long-term adverse reactions in 268 related donors who underwent PBSC mobilization (32). The 15% of donors would have been deferred based on NMDP criteria for unrelated donors due to age (older than 60 years), BMI (at least >40 kg/m^2^), and hypertension (higher than 160/95 mmHg); other medical contraindications to HPC collection included clotting issues, diabetes, or severe heart issues. Of note, the authors detailed the follow-up of donors that would not have been eligible due to NMDP criteria, not reporting an increase in cardiovascular events, autoimmune diseases, or malignancy post-PBSC collection (32). Likewise, in the subsequent follow-up of related donors, we did not report the onset of additional medical conditions in the group of HPC donors with pre-existing benign medical conditions.

WBMT standards (11) recommend that in case of an active cardiac disorder, detailed evaluation by a cardiologist is required, and cardiac risk stratification should be performed by a specialist. Among CVD reported in our study population study, the most frequent pre-existing medical conditions were hypertension. All related donors were classified as ASA-PS <3. In addition, if a donor were considered suitable, we planned an in-depth cardiological evaluation with final approval for apheresis and extracorporeal circulation during the leukapheresis procedure, and an additionally fully anesthesiologic assessment in HPC-M donors, with continuous monitoring of the donor’s vital parameters during and immediately after the collection procedure. Among CVD donors, stem cell collection was performed mainly through apheresis, while only two donors were subjected to BM harvesting; in none of them, we recorded severe adverse events. At the same time, donors suffering from allergic diathesis did not experience side effects during HPC collection, nor patients who received grafts from donors with drug allergies develop an allergy after HSCT.

The WBMT board agreed that HPC donation is typically to be disregarded in donors with systemic multiorgan involvement related to AID; on the other hand, among donors affected by single-organ autoimmune diseases such as Hashimoto thyroiditis, Graves’ disease, pernicious anemia, psoriasis, alopecia areata, or vitiligo, the experts recommended that donation must be deferred only for candidate donors receiving systemic treatment (11). In our institution, all donors with AID were deferred from HPC donations, and a medical history of autoimmune diseases represented the main reason for non-eligibility. The impact on the recipient’s immune reconstitution is not yet known. However, it is widely acknowledged that the pathogenesis of autoimmune disorders is multifactorial, with genetic and environmental factors combining to determine disease onset and evolution. For many autoimmune disorders, adoptive transfer of diseases from the donor to the recipient during allogeneic HSCT has been documented. They include thyroid diseases, type 1 diabetes, immune thrombocytopenia, vitiligo, and psoriasis (38–42).

Large registry-based studies have shown that younger donor age is the most important secondary donor characteristic after HLA matching and has been associated with improved overall and disease-free survival, with a 3% improvement in 2-year survival when a donor 10 years younger is selected (43–45). We did not find a significant correlation between the age of related donors and transplant outcome; the median donor age of our population was 40 years old (range, 16–75), which is usually used as a cutoff for younger versus older donors. Notably, 15 donors were older than 60 years, and three of them also suffered from CVD, in agreement with the increasing prevalence of conditions such as atherosclerotic cardiovascular disorders with aging. Aging is marked by the acquisition of somatic mutations in hematopoietic stem cells due to cumulative genomic DNA damage: this condition is commonly referred to as clonal hematopoiesis of indeterminate potential (CHIP) (46). The proportion of CHIP carriers increases exponentially with age even if mutations within genes including DNMT3A or TET2 can be detected for up to 95% of healthy individuals, with a mean age of 50 years old (47). CHIP is associated with a 0.5%–1% risk per year of leukemia (47). Remarkably, it confers a twofold increase in cardiovascular risk independent of traditional risk factors (46). In fact, CHIP is associated with a pro-inflammatory state that has been linked to coronary artery disease, myocardial infarction, and venous thromboembolic disease (48, 49). In the context of HSCT, the potential transfer of CHIP from donor to patient may bring up concerning implications. An early report first described the transfer of CHIP-mutated hematopoietic stem cells to recipients through HSCT (50). Indeed, these data imply precaution in selecting aging relatives suffering from CVD for donation and suggest that the CHIP screening could be worthwhile in these cases.

Our study exhibits some limitations. First, the short length of the follow-up of the study population requires caution in the interpretation of data. Second, the retrospective design does not allow to reach definite evidence. Finally, recipients were affected by different types of diseases, and the effect of various immunosuppressive regimens was not considered on the GVHD outcome.

Nevertheless, our results provide insights into the related donor selection and suggest that relatives may be accepted for safe HPC donation based on less strict criteria than unrelated donors, offering a lifesaving opportunity for patients whose allogeneic transplants cannot be postponed. Further multicenter studies on larger donor populations are worthy to compare donor selection policies and come to definite conclusions.

## Data availability statement

The raw data supporting the conclusions of this article will be made available by the authors, without undue reservation.

## Ethics statement

The studies involving humans were approved by The Ethics Committee of the Fondazione Policlinico Universitario Agostino Gemelli IRCCS, Largo Agostino Gemelli, 8, 00168, Rome, Italy. The studies were conducted in accordance with the local legislation and institutional requirements. The participants provided their written informed consent to participate in this study. Written informed consent was obtained from the patients for their anonymized information to be published in this article.

## Author contributions

CGV: Writing – original draft, Writing – review & editing. SC: Writing – original draft. FF: Writing – original draft. EM: Writing – original draft. CP: Writing – original draft. PC: Writing – review & editing. SS: Writing – review & editing. LT: Writing – original draft, Writing – review & editing.
